# 3-Amino-1-(3,4-dimeth­oxy­phen­yl)-9,10-dihydro­phenanthrene-2,4-dicarbonitrile

**DOI:** 10.1107/S1600536812011129

**Published:** 2012-03-21

**Authors:** Abdullah M. Asiri, Hassan M. Faidallah, Khalid A. Alamry, Seik Weng Ng, Edward R. T. Tiekink

**Affiliations:** a Chemistry Department, Faculty of Science, King Abdulaziz University, PO Box 80203, Jeddah, Saudi Arabia; b The Center of Excellence for Advanced Materials Research, King Abdulaziz University, Jeddah, PO Box 80203, Saudi Arabia; c Department of Chemistry, University of Malaya, 50603 Kuala Lumpur, Malaysia

## Abstract

In the title compound, C_24_H_19_N_3_O_2_, the partially saturated ring adopts a distorted half-chair conformation with the methyl­ene-C atom closest to the amino­benzene ring lying 0.664 (3) Å out of the plane defined by the five remaining atoms (r.m.s. deviation = 0.1429 Å. The dihedral angle [32.01 (10)°] between the benzene rings on either side of this ring indicates a significant fold in this part of the mol­ecule. The dimeth­oxy-substituted benzene ring is almost orthogonal to the benzene ring to which it is attached [dihedral angle = 72.03 (9)°]. The mol­ecule has been observed previously as the major component of a 1:19 co-crystal with 2-amino-4-(3,4-dimeth­oxy­phen­yl)-5,6-dihydro­benzo[*h*a]quinoline-3-carbonitrile [Asiri *et al.* (2011). *Acta Cryst*. E**67**, o2873–o2873]. Supra­molecular chains with base vector [201] are formed in the crystal structure *via* N—H⋯O hydrogen bonds between the amino H atoms of one mol­ecule inter­acting with the meth­oxy O atoms of a neighbouring mol­ecule. The chains are linked into a three-dimensional architecture by C—H⋯π inter­actions.

## Related literature

For background to the biological activity of related phenanthrene compounds, see: Wang *et al.* (2010 ▶); Rostom *et al.* (2011 ▶). For related structures, see: Asiri *et al.* (2011*a* ▶,*b* ▶); Al-Youbi *et al.* (2012 ▶).
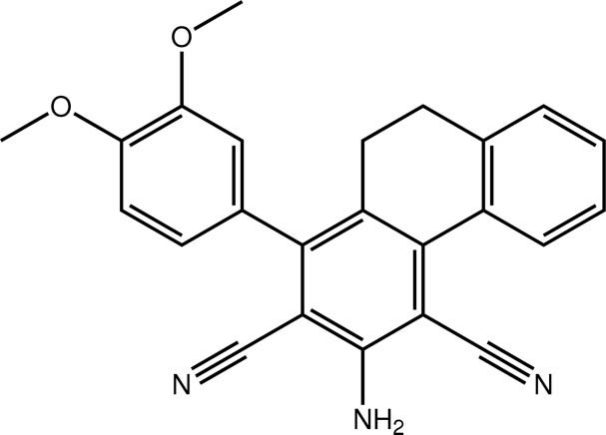


## Experimental

### 

#### Crystal data

C_24_H_19_N_3_O_2_
*M* *_r_* = 381.42
Monoclinic, 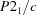

*a* = 8.9360 (7) Å
*b* = 14.5007 (11) Å
*c* = 14.8074 (11) Å
β = 103.471 (8)°
*V* = 1865.9 (2) Å^3^
*Z* = 4
Mo *K*α radiation
μ = 0.09 mm^−1^
*T* = 100 K
0.20 × 0.15 × 0.10 mm

#### Data collection

Agilent SuperNova Dual diffractometer with an Atlas detector
Absorption correction: multi-scan (*CrysAlis PRO*; Agilent, 2011 ▶) *T*_min_ = 0.983, *T*_max_ = 0.991
8105 measured reflections
4272 independent reflections
2851 reflections with *I* > 2σ(*I*)
*R*_int_ = 0.037

#### Refinement

*R*[*F* ^2^ > 2σ(*F* ^2^)] = 0.055
*wR*(*F* ^2^) = 0.136
*S* = 1.03
4272 reflections
270 parameters
H atoms treated by a mixture of independent and constrained refinement
Δρ_max_ = 0.25 e Å^−3^
Δρ_min_ = −0.23 e Å^−3^

### 

Data collection: *CrysAlis PRO* (Agilent, 2011 ▶); cell refinement: *CrysAlis PRO*; data reduction: *CrysAlis PRO*; program(s) used to solve structure: *SHELXS97* (Sheldrick, 2008 ▶); program(s) used to refine structure: *SHELXL97* (Sheldrick, 2008 ▶); molecular graphics: *ORTEP-3* (Farrugia, 1997 ▶) and *DIAMOND* (Brandenburg, 2006 ▶); software used to prepare material for publication: *publCIF* (Westrip, 2010 ▶).

## Supplementary Material

Crystal structure: contains datablock(s) global, I. DOI: 10.1107/S1600536812011129/sj5212sup1.cif

Structure factors: contains datablock(s) I. DOI: 10.1107/S1600536812011129/sj5212Isup2.hkl

Supplementary material file. DOI: 10.1107/S1600536812011129/sj5212Isup3.cml

Additional supplementary materials:  crystallographic information; 3D view; checkCIF report

## Figures and Tables

**Table 1:** Hydrogen-bond geometry (Å, °) *Cg*1 and *Cg*2 are the centroids of the C1–C6 and C17–C22 rings, respectively.

*D*—H⋯*A*	*D*—H	H⋯*A*	*D*⋯*A*	*D*—H⋯*A*
N2—H1⋯O1^i^	0.95 (2)	2.23 (2)	2.921 (2)	129 (2)
N2—H2⋯O2^i^	0.90 (3)	2.28 (3)	2.984 (2)	135 (2)
C24—H24*B*⋯*Cg*1^ii^	0.98	2.78	3.538 (2)	135
C7—H7*A*⋯*Cg*4^iii^	0.99	2.92	3.792 (2)	147

Symmetry codes: (i) 
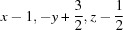
; (ii) 
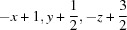
; (iii) 
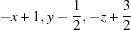
.

**Fig. 1.**
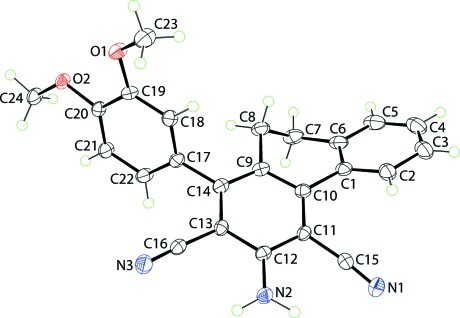
The molecular structure of (I) showing the atom-labelling scheme and displacement ellipsoids at the 50% probability level.

**Fig. 2.**
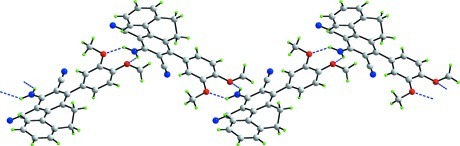
The supramolecular chain in (I) sustained by N—H···O hydrogen bonds shown as blue dashed lines.

**Fig. 3.**
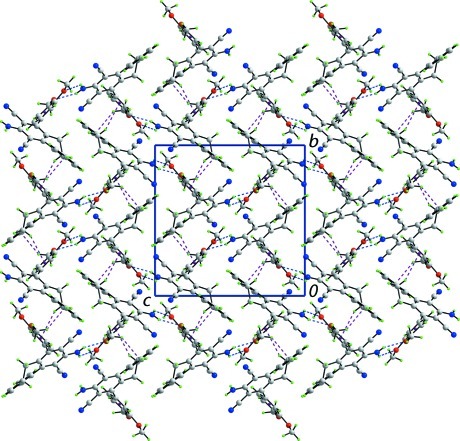
A view in projection down the a axis of the unit-cell contents of (I). The N—H···O hydrogen bonds and C—H···π interactions are shown as blue and purple dashed lines, respectively.

## supplementary crystallographic information

### Comment

The X-ray crystallographic investigation of the title compound, 3-amino-1-(3,4-dimethoxyphenyl)-9,10-dihydrophenanthrene-2,4-dicarbonitrile (I), was motivated by reports of the biological activity of related compounds (Wang *et al.*, 2010; Rostom *et al.*, 2011) and allied crystal structure investigations (Asiri *et al.*, 2011*a*; Al-Youbi *et al.*, 2012). The molecule of (I) has been observed previously in its 1/19 co-crystal with 2-amino-4-(3,4-dimethoxyphenyl)-5,6-dihydrobenzo[*h*a]quinoline-3-carbonitrile (Asiri *et al.*, 2011*b*).

In (I), Fig. 1, the partially saturated ring adopts a distorted twisted half chair conformation with the C2 atom lying 0.664 (3) Å out of the plane defined by the five remaining atoms [r.m.s. deviation = 0.1429 Å; maximum deviations = 0.1733 (11) Å for the C9 atom and -0.2097 (14) Å for the C10 atom]. The dihedral angle between the benzene rings on either side of this ring = 32.01 (10)°, indicating a significant fold in this part of the molecule. The dimethoxy-substituted benzene ring is almost normal to the plane of the benzene ring to which it is attached, forming a dihedral angle of 72.03 (9)° The O1-and O2-methoxy substituents are each slightly twisted out of the plane of the benzene ring to which they are attached as seen in the values of the C23—O1—C19—C18 and C24—O2—C20—C21 torsion angles of -13.7 (3) and -5.0 (3)°, respectively; they lie to opposite sides of the plane through the benzene ting.

The most prominent feature in the crystal packing is the formation of N—H···O hydrogen bonds whereby the amino-H atoms are connected to the two methoxy-O atoms of a neighbouring molecule leading to a seven-membered {···HNH···OC_2_O} synthon linked into twisted supramolecular chains, Fig. 2 and Table 1; the base vector is along [2 0 1]. Clearly, the presence of two oxygen atoms in (I), is sufficient to disrupt the normally formed N—H···N hydrogen bonds between centrosymmetrically related molecules leading to to 12-membered {···HNC_3_N}_2_ synthons (Asiri *et al.*, 2011*a*; Asiri *et al.*, 2011*b*). Supramolecular chains are sustained in a three-dimensional architecture by C—H···π interactions, Fig. 3 and Table 1.

### Experimental

A mixture of 3,4-dimethoxybenzaldehyde (1.66 g, 0.01 mmol), 1-tetralone (1.46 g, 0.01 mmol), malononitrile (0.66 g, 0.01 mmol) and ammonium acetate (6.2 g, 0.08 mmol) in absolute ethanol (50 ml) was refluxed for 6 h. The reaction mixture was allowed to cool. The precipitate that formed was filtered, washed with water, dried and recrystallized from ethanol. Yield: 69%, *M*. pt. 533–535 K.

### Refinement

Carbon-bound H-atoms were placed in calculated positions [C—H = 0.95 to 0.99 Å, *U*_iso_(H) = 1.2 to 1.5*U*_eq_(C)] and were included in the refinement in the riding model approximation. The N—H atoms were located in a difference Fourier map, and were refined with a distance restraint of N—H = 0.88±0.01 Å; their *U*_iso_ values were refined. Owing to poor agreement, the (1 15 5) reflection was omitted from the final cycles of refinement.

### Crystal data

**Table:**

C_24_H_19_N_3_O_2_	*F*(000) = 800
*M**_r_* = 381.42	*D*_x_ = 1.358 Mg m^−^^3^
Monoclinic, *P*2_1_/*c*	Mo *K*α radiation, λ = 0.71073 Å
Hall symbol: -P 2ybc	Cell parameters from 2185 reflections
*a* = 8.9360 (7) Å	θ = 2.4–27.5°
*b* = 14.5007 (11) Å	µ = 0.09 mm^−^^1^
*c* = 14.8074 (11) Å	*T* = 100 K
β = 103.471 (8)°	Chip, orange
*V* = 1865.9 (2) Å^3^	0.20 × 0.15 × 0.10 mm
*Z* = 4	

### Data collection

**Table:**

Agilent SuperNova Dual diffractometer with an Atlas detector	4272 independent reflections
Radiation source: SuperNova (Mo) X-ray Source	2851 reflections with *I* > 2σ(*I*)
Mirror monochromator	*R*_int_ = 0.037
Detector resolution: 10.4041 pixels mm^-1^	θ_max_ = 27.6°, θ_min_ = 2.7°
ω scan	*h* = −11→11
Absorption correction: multi-scan (*CrysAlis PRO*; Agilent, 2011)	*k* = −18→17
*T*_min_ = 0.983, *T*_max_ = 0.991	*l* = −11→19
8105 measured reflections	

### Refinement

**Table:**

Refinement on *F*^2^	Primary atom site location: structure-invariant direct methods
Least-squares matrix: full	Secondary atom site location: difference Fourier map
*R*[*F*^2^ > 2σ(*F*^2^)] = 0.055	Hydrogen site location: inferred from neighbouring sites
*wR*(*F*^2^) = 0.136	H atoms treated by a mixture of independent and constrained refinement
*S* = 1.03	*w* = 1/[σ^2^(*F*_o_^2^) + (0.0526*P*)^2^ + 0.2552*P*] where *P* = (*F*_o_^2^ + 2*F*_c_^2^)/3
4272 reflections	(Δ/σ)_max_ = 0.001
270 parameters	Δρ_max_ = 0.25 e Å^−^^3^
0 restraints	Δρ_min_ = −0.23 e Å^−^^3^

### Fractional atomic coordinates and isotropic or equivalent isotropic displacement parameters (Å^2^)

**Table:**

	*x*	*y*	*z*	*U*_iso_*/*U*_eq_	
O1	0.69410 (14)	0.85996 (10)	0.89152 (10)	0.0255 (4)	
O2	0.92377 (14)	0.80208 (9)	0.82524 (9)	0.0215 (3)	
N1	−0.21440 (18)	0.47271 (12)	0.60427 (12)	0.0271 (4)	
N2	−0.0035 (2)	0.62438 (13)	0.51881 (12)	0.0243 (4)	
N3	0.31457 (19)	0.75869 (13)	0.50677 (13)	0.0278 (4)	
C1	0.1055 (2)	0.47341 (14)	0.82665 (13)	0.0231 (5)	
C2	−0.0456 (2)	0.47157 (15)	0.83797 (15)	0.0289 (5)	
H2A	−0.1234	0.5058	0.7968	0.035*	
C3	−0.0831 (3)	0.42019 (16)	0.90882 (16)	0.0347 (6)	
H3	−0.1859	0.4194	0.9160	0.042*	
C4	0.0298 (3)	0.37015 (16)	0.96890 (16)	0.0393 (6)	
H4	0.0038	0.3338	1.0164	0.047*	
C5	0.1807 (3)	0.37300 (15)	0.95975 (15)	0.0350 (6)	
H5	0.2576	0.3388	1.0014	0.042*	
C6	0.2208 (2)	0.42536 (15)	0.89025 (14)	0.0279 (5)	
C7	0.3846 (2)	0.43614 (15)	0.88204 (15)	0.0313 (5)	
H7A	0.4029	0.3973	0.8307	0.038*	
H7B	0.4561	0.4162	0.9403	0.038*	
C8	0.4127 (2)	0.53735 (15)	0.86299 (14)	0.0273 (5)	
H8A	0.3967	0.5760	0.9151	0.033*	
H8B	0.5200	0.5459	0.8573	0.033*	
C9	0.3017 (2)	0.56644 (14)	0.77347 (13)	0.0215 (4)	
C10	0.1525 (2)	0.52829 (14)	0.75366 (13)	0.0207 (4)	
C11	0.0532 (2)	0.54618 (13)	0.66663 (13)	0.0182 (4)	
C12	0.0953 (2)	0.60554 (13)	0.60093 (13)	0.0189 (4)	
C13	0.2426 (2)	0.64665 (13)	0.62557 (13)	0.0186 (4)	
C14	0.3445 (2)	0.62742 (13)	0.71120 (13)	0.0198 (4)	
C15	−0.0950 (2)	0.50286 (14)	0.63642 (14)	0.0221 (4)	
C16	0.2857 (2)	0.70935 (14)	0.56101 (14)	0.0210 (4)	
C17	0.4968 (2)	0.67539 (14)	0.73596 (13)	0.0199 (4)	
C18	0.5213 (2)	0.74481 (14)	0.80346 (14)	0.0215 (5)	
H18	0.4402	0.7623	0.8315	0.026*	
C19	0.6633 (2)	0.78845 (13)	0.82984 (13)	0.0193 (4)	
C20	0.7852 (2)	0.75935 (13)	0.79148 (13)	0.0182 (4)	
C21	0.7588 (2)	0.69338 (14)	0.72295 (14)	0.0241 (5)	
H21	0.8394	0.6761	0.6944	0.029*	
C22	0.6146 (2)	0.65146 (15)	0.69485 (14)	0.0249 (5)	
H22	0.5977	0.6062	0.6471	0.030*	
C23	0.5626 (2)	0.90343 (16)	0.91355 (16)	0.0333 (6)	
H23A	0.5969	0.9532	0.9583	0.050*	
H23B	0.5047	0.8578	0.9405	0.050*	
H23C	0.4963	0.9289	0.8568	0.050*	
C24	1.0509 (2)	0.76905 (15)	0.79032 (15)	0.0257 (5)	
H24A	1.1434	0.8047	0.8181	0.039*	
H24B	1.0274	0.7762	0.7227	0.039*	
H24C	1.0688	0.7038	0.8063	0.039*	
H1	−0.105 (3)	0.5996 (17)	0.5047 (17)	0.044 (7)*	
H2	0.028 (3)	0.6568 (18)	0.4750 (18)	0.040 (7)*	

### Atomic displacement parameters (Å^2^)

**Table:**

	*U*^11^	*U*^22^	*U*^33^	*U*^12^	*U*^13^	*U*^23^
O1	0.0178 (7)	0.0281 (8)	0.0307 (8)	0.0001 (6)	0.0057 (6)	−0.0119 (7)
O2	0.0141 (6)	0.0248 (8)	0.0260 (7)	−0.0022 (5)	0.0054 (5)	−0.0058 (6)
N1	0.0207 (9)	0.0277 (10)	0.0327 (10)	−0.0014 (7)	0.0060 (7)	0.0010 (8)
N2	0.0186 (9)	0.0298 (11)	0.0222 (9)	−0.0030 (7)	−0.0002 (7)	0.0048 (8)
N3	0.0257 (9)	0.0278 (10)	0.0300 (10)	−0.0031 (7)	0.0065 (8)	0.0015 (9)
C1	0.0304 (11)	0.0185 (11)	0.0196 (10)	−0.0063 (8)	0.0040 (8)	−0.0041 (9)
C2	0.0353 (12)	0.0265 (12)	0.0266 (11)	−0.0058 (9)	0.0111 (9)	−0.0031 (10)
C3	0.0497 (15)	0.0289 (13)	0.0306 (12)	−0.0118 (10)	0.0193 (11)	−0.0060 (10)
C4	0.0641 (17)	0.0315 (14)	0.0255 (12)	−0.0142 (12)	0.0170 (11)	−0.0022 (11)
C5	0.0537 (15)	0.0264 (13)	0.0213 (11)	−0.0054 (11)	0.0016 (10)	0.0002 (10)
C6	0.0400 (13)	0.0228 (12)	0.0179 (10)	−0.0055 (9)	0.0008 (9)	−0.0011 (9)
C7	0.0355 (12)	0.0286 (13)	0.0232 (11)	0.0008 (9)	−0.0063 (9)	0.0025 (10)
C8	0.0278 (11)	0.0302 (12)	0.0203 (10)	−0.0025 (9)	−0.0020 (8)	−0.0014 (10)
C9	0.0222 (10)	0.0198 (11)	0.0200 (10)	0.0011 (8)	−0.0001 (8)	−0.0023 (9)
C10	0.0229 (10)	0.0191 (11)	0.0196 (10)	−0.0005 (8)	0.0038 (8)	−0.0020 (9)
C11	0.0161 (9)	0.0168 (10)	0.0221 (9)	−0.0009 (7)	0.0049 (7)	−0.0037 (8)
C12	0.0163 (9)	0.0192 (10)	0.0202 (9)	0.0017 (7)	0.0025 (7)	−0.0024 (8)
C13	0.0164 (9)	0.0177 (10)	0.0221 (10)	−0.0003 (7)	0.0055 (7)	−0.0015 (8)
C14	0.0163 (9)	0.0206 (10)	0.0223 (10)	0.0021 (8)	0.0036 (8)	−0.0048 (9)
C15	0.0241 (10)	0.0220 (11)	0.0215 (10)	0.0001 (8)	0.0080 (8)	0.0011 (9)
C16	0.0165 (10)	0.0221 (11)	0.0234 (10)	−0.0007 (8)	0.0026 (8)	−0.0029 (9)
C17	0.0155 (9)	0.0217 (10)	0.0206 (10)	0.0007 (8)	0.0001 (7)	0.0006 (9)
C18	0.0152 (9)	0.0264 (11)	0.0229 (10)	0.0026 (8)	0.0042 (8)	−0.0011 (9)
C19	0.0188 (10)	0.0190 (10)	0.0185 (9)	0.0019 (8)	0.0009 (7)	−0.0034 (8)
C20	0.0132 (9)	0.0200 (10)	0.0203 (10)	0.0014 (7)	0.0013 (7)	0.0023 (8)
C21	0.0177 (10)	0.0292 (12)	0.0266 (11)	−0.0009 (8)	0.0078 (8)	−0.0060 (9)
C22	0.0206 (10)	0.0298 (12)	0.0232 (10)	−0.0008 (8)	0.0030 (8)	−0.0078 (9)
C23	0.0241 (11)	0.0366 (14)	0.0410 (13)	0.0009 (9)	0.0109 (9)	−0.0171 (11)
C24	0.0177 (10)	0.0287 (12)	0.0323 (11)	−0.0027 (8)	0.0087 (8)	−0.0074 (10)

### Geometric parameters (Å, º)

**Table:**

O1—C19	1.367 (2)	C8—H8B	0.9900
O1—C23	1.437 (2)	C9—C14	1.394 (3)
O2—C20	1.371 (2)	C9—C10	1.410 (3)
O2—C24	1.436 (2)	C10—C11	1.408 (2)
N1—C15	1.149 (2)	C11—C12	1.414 (3)
N2—C12	1.354 (2)	C11—C15	1.440 (3)
N2—H1	0.95 (2)	C12—C13	1.413 (3)
N2—H2	0.90 (3)	C13—C14	1.406 (2)
N3—C16	1.149 (3)	C13—C16	1.435 (3)
C1—C2	1.400 (3)	C14—C17	1.496 (3)
C1—C6	1.408 (3)	C17—C22	1.377 (3)
C1—C10	1.479 (3)	C17—C18	1.400 (3)
C2—C3	1.390 (3)	C18—C19	1.390 (3)
C2—H2A	0.9500	C18—H18	0.9500
C3—C4	1.384 (3)	C19—C20	1.405 (3)
C3—H3	0.9500	C20—C21	1.374 (3)
C4—C5	1.387 (3)	C21—C22	1.398 (3)
C4—H4	0.9500	C21—H21	0.9500
C5—C6	1.391 (3)	C22—H22	0.9500
C5—H5	0.9500	C23—H23A	0.9800
C6—C7	1.504 (3)	C23—H23B	0.9800
C7—C8	1.526 (3)	C23—H23C	0.9800
C7—H7A	0.9900	C24—H24A	0.9800
C7—H7B	0.9900	C24—H24B	0.9800
C8—C9	1.518 (2)	C24—H24C	0.9800
C8—H8A	0.9900		
			
C19—O1—C23	115.90 (15)	C12—C11—C15	115.13 (16)
C20—O2—C24	116.20 (15)	N2—C12—C13	121.33 (19)
C12—N2—H1	120.6 (15)	N2—C12—C11	121.24 (17)
C12—N2—H2	120.2 (15)	C13—C12—C11	117.39 (16)
H1—N2—H2	119 (2)	C14—C13—C12	121.20 (18)
C2—C1—C6	119.1 (2)	C14—C13—C16	120.53 (17)
C2—C1—C10	122.94 (18)	C12—C13—C16	118.26 (16)
C6—C1—C10	117.84 (19)	C9—C14—C13	120.14 (17)
C3—C2—C1	120.6 (2)	C9—C14—C17	120.47 (16)
C3—C2—H2A	119.7	C13—C14—C17	119.35 (18)
C1—C2—H2A	119.7	N1—C15—C11	173.3 (2)
C4—C3—C2	119.9 (2)	N3—C16—C13	177.17 (19)
C4—C3—H3	120.0	C22—C17—C18	119.25 (18)
C2—C3—H3	120.0	C22—C17—C14	121.31 (18)
C3—C4—C5	120.0 (2)	C18—C17—C14	119.43 (18)
C3—C4—H4	120.0	C19—C18—C17	120.58 (19)
C5—C4—H4	120.0	C19—C18—H18	119.7
C4—C5—C6	120.8 (2)	C17—C18—H18	119.7
C4—C5—H5	119.6	O1—C19—C18	124.16 (18)
C6—C5—H5	119.6	O1—C19—C20	116.35 (16)
C5—C6—C1	119.4 (2)	C18—C19—C20	119.49 (18)
C5—C6—C7	122.66 (19)	O2—C20—C21	124.66 (18)
C1—C6—C7	117.94 (19)	O2—C20—C19	115.86 (17)
C6—C7—C8	108.65 (18)	C21—C20—C19	119.47 (17)
C6—C7—H7A	110.0	C20—C21—C22	120.66 (19)
C8—C7—H7A	110.0	C20—C21—H21	119.7
C6—C7—H7B	110.0	C22—C21—H21	119.7
C8—C7—H7B	110.0	C17—C22—C21	120.35 (19)
H7A—C7—H7B	108.3	C17—C22—H22	119.8
C9—C8—C7	109.05 (16)	C21—C22—H22	119.8
C9—C8—H8A	109.9	O1—C23—H23A	109.5
C7—C8—H8A	109.9	O1—C23—H23B	109.5
C9—C8—H8B	109.9	H23A—C23—H23B	109.5
C7—C8—H8B	109.9	O1—C23—H23C	109.5
H8A—C8—H8B	108.3	H23A—C23—H23C	109.5
C14—C9—C10	120.23 (16)	H23B—C23—H23C	109.5
C14—C9—C8	121.98 (17)	O2—C24—H24A	109.5
C10—C9—C8	117.78 (18)	O2—C24—H24B	109.5
C11—C10—C9	118.79 (18)	H24A—C24—H24B	109.5
C11—C10—C1	122.85 (17)	O2—C24—H24C	109.5
C9—C10—C1	118.33 (16)	H24A—C24—H24C	109.5
C10—C11—C12	122.02 (16)	H24B—C24—H24C	109.5
C10—C11—C15	122.79 (18)		
			
C6—C1—C2—C3	−2.5 (3)	C11—C12—C13—C14	1.5 (3)
C10—C1—C2—C3	−178.78 (19)	N2—C12—C13—C16	−0.1 (3)
C1—C2—C3—C4	−0.1 (3)	C11—C12—C13—C16	−178.12 (18)
C2—C3—C4—C5	1.5 (3)	C10—C9—C14—C13	−4.1 (3)
C3—C4—C5—C6	−0.4 (3)	C8—C9—C14—C13	174.73 (19)
C4—C5—C6—C1	−2.2 (3)	C10—C9—C14—C17	173.87 (19)
C4—C5—C6—C7	175.3 (2)	C8—C9—C14—C17	−7.3 (3)
C2—C1—C6—C5	3.6 (3)	C12—C13—C14—C9	0.4 (3)
C10—C1—C6—C5	−179.94 (18)	C16—C13—C14—C9	−179.99 (19)
C2—C1—C6—C7	−174.09 (19)	C12—C13—C14—C17	−177.60 (18)
C10—C1—C6—C7	2.4 (3)	C16—C13—C14—C17	2.0 (3)
C5—C6—C7—C8	−135.7 (2)	C9—C14—C17—C22	107.8 (2)
C1—C6—C7—C8	41.9 (2)	C13—C14—C17—C22	−74.2 (3)
C6—C7—C8—C9	−59.5 (2)	C9—C14—C17—C18	−71.0 (3)
C7—C8—C9—C14	−143.2 (2)	C13—C14—C17—C18	106.9 (2)
C7—C8—C9—C10	35.7 (3)	C22—C17—C18—C19	−0.9 (3)
C14—C9—C10—C11	5.8 (3)	C14—C17—C18—C19	177.97 (18)
C8—C9—C10—C11	−173.11 (18)	C23—O1—C19—C18	−13.7 (3)
C14—C9—C10—C1	−172.40 (19)	C23—O1—C19—C20	165.87 (18)
C8—C9—C10—C1	8.7 (3)	C17—C18—C19—O1	176.45 (17)
C2—C1—C10—C11	−31.5 (3)	C17—C18—C19—C20	−3.1 (3)
C6—C1—C10—C11	152.19 (19)	C24—O2—C20—C21	−5.0 (3)
C2—C1—C10—C9	146.6 (2)	C24—O2—C20—C19	176.54 (17)
C6—C1—C10—C9	−29.7 (3)	O1—C19—C20—O2	4.2 (2)
C9—C10—C11—C12	−3.9 (3)	C18—C19—C20—O2	−176.20 (17)
C1—C10—C11—C12	174.20 (19)	O1—C19—C20—C21	−174.28 (17)
C9—C10—C11—C15	173.50 (19)	C18—C19—C20—C21	5.3 (3)
C1—C10—C11—C15	−8.4 (3)	O2—C20—C21—C22	178.03 (18)
C10—C11—C12—N2	−177.79 (19)	C19—C20—C21—C22	−3.6 (3)
C15—C11—C12—N2	4.6 (3)	C18—C17—C22—C21	2.6 (3)
C10—C11—C12—C13	0.3 (3)	C14—C17—C22—C21	−176.20 (18)
C15—C11—C12—C13	−177.29 (18)	C20—C21—C22—C17	−0.4 (3)
N2—C12—C13—C14	179.56 (19)		

### Hydrogen-bond geometry (Å, º)

Cg1 and Cg2 are the centroids of the C1–C6 and C17–C22 rings, respectively.

**Table:**

*D*—H···*A*	*D*—H	H···*A*	*D*···*A*	*D*—H···*A*
N2—H1···O1^i^	0.95 (2)	2.23 (2)	2.921 (2)	129 (2)
N2—H2···O2^i^	0.90 (3)	2.28 (3)	2.984 (2)	135 (2)
C24—H24*B*···*Cg*1^ii^	0.98	2.78	3.538 (2)	135
C7—H7*A*···*Cg*4^iii^	0.99	2.92	3.792 (2)	147

Symmetry codes: (i) *x*−1, −*y*+3/2, *z*−1/2; (ii) −*x*+1, *y*+1/2, −*z*+3/2; (iii) −*x*+1, *y*−1/2, −*z*+3/2.
